# Factors considered by undergraduate medical students when selecting specialty of their future careers

**DOI:** 10.11604/pamj.2015.20.102.4715

**Published:** 2015-02-04

**Authors:** Awad Ali Mohamed Ahmed Alawad, Waleed Shabeer Khan, Yousif Mohammed Abdelrazig, Yamin Ibrahim Elzain, Hassan Osman khalil, Omer Bakri Elsayed Ahmed, Omeralfaroug Ahmed Ibrahim Adam

**Affiliations:** 1 Assistant Professor of Surgery, University of Medical Sciences and Technology, Khartoum, Sudan; 2 MBBS, University of Medical Sciences and Technology, Khartoum, Sudan

**Keywords:** Medical education, future career, specialty choice, career choice, medical students, Sudan

## Abstract

**Introduction:**

Medical students are the source of a country's physicians. Determining how medical students select their areas of specialization is the key to achieve a balanced distribution of doctors among all specialties. The objective is to identify the number of medical students who have decided their postgraduate specialty career, their career specialties preference, and factors that may influence their decision to select a particular specialty.

**Methods:**

A facility based cross-sectional study was conducted in September 2013 at Faculty of Medicine, University of Medical Sciences and Technology, Khartoum, Sudan. A self-administered semi-structured questionnaire comprising demographic data and questions about future specialties preferences and factors influencing those preferences was distributed to 887 male and female students, (from first to fifth academic years) recruited in the study.

**Results:**

Response rate was 73% with 647 questionnaires collected, out of 887 eligible medical students. Of the returned questionnaires, 604 were valid. The majority of students (541, 89.6%) have chosen a specialty. Surgery, medicine, paediatrics and obstetrics and gynecology were the most selected specialties. The least selected specialty was anaesthesiology. A significant association was found between gender and specialty choice using Chi-square test (p = 0.00). There was no association between undergraduate level and specialty choice (p = 0.633). The most common reason for choosing a specific specialty was “Personal Interest” (215, 39.7%) followed by being “Helpful to the community” (144, 26.6%).

**Conclusion:**

Surgery, medicine, paediatrics and obstetrics and gynecology were the most selected specialties.

## Introduction

After graduating from medical school, medical students have to decide a career path to pursue for further specialization. The future career specialties chosen by medical students and the factors involved in making these choices are of great significance for balanced distribution of doctors in the different specialties and for planning of the workforce of healthcare services. During the course of their study in medical school, undergraduates are exposed to a wide range of medical specialties and by the time they graduate, most students will have sampled many of the different areas of medical practice. As a result, it is presumed that students do not decide on a specialty until after they have graduated from medical school [1]. However, medical school entrants, and even those applying for medical school, usually have strong preference for and against some medical specialties [2, 3]. Therefore, there is strong evidence that students decide their future medical career during or even before medical school [4, 5].

This choice, however, can change during the course of medical school [6–9]. Many factors influence the medical students’ choice of future career [9–12]. Generally, these factors are classified into two groups; intrinsic factors, for example those related to personal attributes and preferences, and extrinsic factors like those related to work environment [6]. The impact of these factors varies from setting to setting. In United Kingdom, other peoples’ perception of the job and fewer practice hours were considered the most important factors by students, whereas in Turkey, financials and prestige were more important [13, 14]. On the other hand, students in Jordan considered the intellectual content of the specialty and the individual's competencies most influential while a personal interest in the specialty was the deriving factor for students in Kingdom of Saudi Arabia, Taiwan, Pakistan and India [6, 9, 15–17]. Gender is another important factor that influences career choice. It affects not only the career chosen, but also the reasons that contribute to this choice. It is suggested that males and females usually prefer careers that are consistent with their gender “schemas”; females usually choose paediatrics and obstetrics & gynecology and men choose surgical specialties [16]. This study aimed to determine the number of students that have chosen a career, their career preference and the factors influencing those choices in medical students attending the University of Medical Sciences and Technology.

## Methods

A facility-based cross-sectional study was conducted in September 2013 at Faculty of Medicine, University of Medical Sciences and Technology, Khartoum, Sudan. A self-administered, semi-structured questionnaire comprising demographic data and questions about future specialties preferences and factors influencing those preferences was distributed to 887 male and female students, (from first to fifth academic years) recruited in the study. Total coverage of the students was done to collect data and questionnaires were distributed to all of the students in faculty of medicine, University of Medical Sciences and technology. Data was checked for completeness, consistency and range. IBM SPSS version 20 was used to analyze the data. Descriptive frequency analysis was done for all variables. Chi-square test was used to assess the association between gender and specialty preference. Bivariate correlation using spearman correlation test was used to assess the association between undergraduate level and specialty preference. All summary statistics are stated with 95% confidence limits. The purpose and nature of the study were fully explained to the students and a verbal consent was obtained from each participant. The potential participants were clearly assured that their participation in this study is voluntary and that they could withdraw at any stage and that any data obtained would be treated confidentially and for the purpose of the research only.

## Results

The total number of medical students at University of Medical Sciences and Technology is 887. A total of 887 questionnaires were handed out, of these 647 questionnaires were returned (response rate of 73%). Of the returned questionnaires 604 were valid and considered. Table 1 shows the general characteristics of the valid responses. The majority of our respondents were females (371, 61.4%) and 233 students were males (38.6%). Most of the respondents (592, 98%) were unmarried while only 12 (2%) were married. A total of 445 students (73.7%) were at preclinical level (1^st^, 2^nd^ and 3^rd^ years) and 159 students (26.3%) were at clinical level (4^th^ and 5^th^ years). The majority of students (501, 83%) had a family member in the medical field while only 103 students (17%) did not.

**Table 1:** The general characteristics and other related variables of present study participants

Characteristics	No of Students (%)	
Gender	Male	233 (38.6)
	Female	371 (61.4)
Marital status	Single	592 (98)
	Married	12 (2)
UG Level	1st year	157 (26)
	2nd year	145 (24)
	3rd year	143 (23.7)
	4th year	97 (16.1)
	5th year	62 (10.3)
Family in Medical Field	Yes	501 (82.9)
	No	103 (17.1)
Information about specialties choice	Yes	534 (88.4)
	No	70 (11.6)
Source of information	Friends	75 (12.4)
	Relatives	282 (46.7)
	Internet	85 (14.1)
	Books	34 (5.6)
	Workshop	11 (1.8)
	Others	52 (8.6)
Have a specialty preference	Yes	541 (89.6)
	No	63 (10.4)

**Figure 1:**
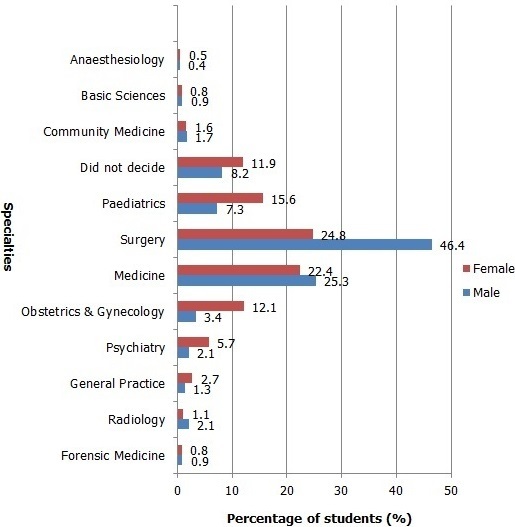
All the specialties chosen by male and female respondents with their corresponding percentages. The percentages are within the gender

**Figure 2:**
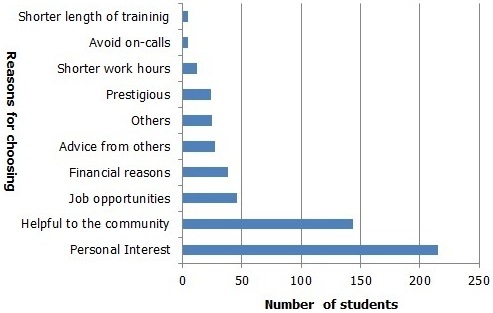
The reasons for choosing a specialty

Of the respondents 539 students (89.2%) had background information about the specialties they have chosen while 65 students (10.8%) did not. The most common source of this background information was “Relatives” (282, 46.7%) followed by “Internet” (85, 14.1%), “Friends” (75, 12.4%), “Others” (52, 8.6%), “Books” (34, 5.6%) and “Workshop” 11 (1.8%). Most of the students have decided upon a specialty (541, 89.6%) while 63 (10.4%) did not. Figure 1 shows all specialties chosen by the respondents. Chi-square test demonstrated significant relation between gender and future specialties chosen (p = 0.00). Among females the most chosen specialty was Surgery (92, 24.8%) and the most chosen specialty by male students was Surgery as well (108, 46.4%). Bivariate correlation using spearman correlation test showed no association between undergraduate level and the future specialty chosen (p = 0.633). Table 2 shows the future specialties chosen by students at pre-clinical and clinical level. Off those who have chosen a specialty the most common reason was “Personal Interest” (215, 39.7%) followed by “Helpful to the community” (144, 26.6%), “Job opportunities” (46, 8.5%) and “Financial reasons” (38, 7.0%). Figure 2 shows the reasons for choosing a specialty with their corresponding frequencies.

**Table 2:** The future specialties chosen by students at pre-clinical and clinical level and their corresponding frequencies and percentages

	Undergraduate Level		
Specialty	Pre-clinical n (%)	Clinical n (%)	Total
Surgery	164 (36.9)	36 (22.6)**	200
Medicine	95 (21.3)	47 (29.6)	142
Paediatrics	55 (12.4)	20 (12.6)	75
Did not decide	34 (7.6)	29 (18.2)	63
Obstetrics & Gynecology	37 (8.3)	16 (10.1)	53
Psychiatry	24 (5.4)	2 (1.3)	26
General Practice	13 (2.9)	0 (0)	13
Community Medicine	8 (1.8)	2 (1.3)	10
Radiology	3 (0.7)	6 (3.8)	9
Forensic Medicine	5 (1.1)	0 (0)	5
Basic Sciences	4 (0.9)	1 (0.6)	5
Anaesthesiology	3 (0.7)	0 (0)	3
Total	445 (100)	159 (100)	604

Pre-clinical: number and percentage of students from total no of students at pre-clinical level; Clinical: number and percentage of students from total no of students at clinical level

## Discussion

The future career specialties preferred and the factors involved in making these choices by medical students are of great significance for balanced distribution of doctors in different specialties. The total number of students enrolled at the University of Medical Sciences and Technology is 887. All of these students were given questionnaires, 647 of questionnaires were answered and only 604 of the questionnaires answered were valid and considered (Table 1). The majority of students (89.6%) students have decided on a specialty. Surgery, medicine, paediatrics and obstetrics and gynecology (in order of their frequency) were the most selected specialties in our study and this is similar to the findings of studies done in Jordan and Greece [9, 12, 18]. The single most popular specialty was surgery and this is in line with the international trend [6, 17, 18].

The least selected specialty was anesthesiology which is consistent with other studies done in the Kingdom of Saudi Arabia and Gambia [6, 8]. This could be explained by minimal exposure of students to anesthesiology as it has low weight in the students’ curriculum compared to other specialties. Another specialty that was not popular with the students was general practice. This low level of interest in general practice is consistent with other studies [18, 19]. A possible explanation for this is the low weight of general practice in the curriculum. Furthermore, general practice is not well established in the country and the students might not see good future job opportunities in this specialty. The distribution of the different specialties selected may be based on multiple causes and influences. One aspect of the specialty selection is the students’ information about the specialty. Out of those questioned 539 students (89.6%) claimed they had background information about specialties, while 63 students (10.4%) said they did not. The most common source of this background information was “Relatives” (282, 46.7%). This indicates that having a relative in the medical field might somehow influence the selection of future specialty. However, such a conclusion cannot be drawn from this study as it was not specifically tested for and further research is needed to support it. The least selected source of this background information was “Workshops” (11, 2%) probably because of the scarcity of such programs in the country.

Many studies have demonstrated an association between gender and choice of future specialty [9, 13]. This association was also found in our study as demonstrated by the Chi-square test (p = 0.0). This difference can be seen in the choices of the students; for instance surgery was the most chosen specialty by both males and females, however, 46.4% of males chose it compared to 24.8% of females. The percentage of the males is two times that of the females. Another example is paediatrics, which was chosen by 7.3% of males compared to 15.6% the females. Most of us in society receive our influence from our surroundings; medical students selecting specialties are no different. As well as the simple fact that students don't have experience to legally practice these specialties so of course the influence towards selection has to be from an environmental source. Many studies have demonstrated a difference in the choices of students in different undergraduate levels [6, 7, 9]. However, bivariate correlation using spearman correlation test showed no association between undergraduate level and the future specialty chosen (p = 0.633) in our study.

The most common reason for choosing a specialty was “Personal Interest” (215, 39.7%) (Figure 2). This is similar to the results of studies in Kingdom of Saudi Arabia, Pakistan and Taiwan [6, 15, 16]. However, other studies showed different findings; financials and prestige were the deriving factors for choosing a specialty in Turkey while in Jordan, it was the intellectual content of the specialty and the individual's competencies [9, 14]. The students’ choice of personal interest as the most influencing reason for choosing a specialty somewhat makes sense because the interest in a topic is what drives most students to pursue study or practice. Although one would assume that the major reasons for selecting specialties would be financial, to avoid on-calls, job opportunities, and shorter work hours. This might be due to the fact that medical students once again have no experience working and are oblivious to the stress and perils associated with working in the medical field. A limitation of this study is that it was done in a single private medical school and hence some of our findings might not represent the career preferences of all medical students in the country. However, it may still be useful in assessing the future career choices of medical students. Never the less, more such studies are needed to get the complete picture of medical students’ career preferences and the factors influencing them in Sudan.

## Conclusion

Surgery, medicine, paediatrics and obstetrics and gynecology were the most selected specialties by the students while anaesthesiology was the least chosen specialty. A personal interest was the most common reason for choosing a specialty in the University of Medical Sciences and technology.
